# Active Fovea-Based Vision Through Computationally-Effective Model-Based Prediction

**DOI:** 10.3389/fnbot.2018.00076

**Published:** 2018-12-14

**Authors:** Emmanuel Daucé

**Affiliations:** Ecole Centrale de Marseille, INSERM, Institut de Neurosciences des Systèmes, Aix Marseille Université, Marseille, France

**Keywords:** intrinsic motivation, foveated vision, saccadic eye movements, active inference, information gain, convolutional neural networks (CNN), active vision

## Abstract

What motivates an action in the absence of a definite reward? Taking the case of visuomotor control, we consider a minimal control problem that is how select the next saccade, in a sequence of discrete eye movements, when the final objective is to better interpret the current visual scene. The visual scene is modeled here as a partially-observed environment, with a generative model explaining how the visual data is shaped by action. This allows to interpret different action selection metrics proposed in the literature, including the Salience, the Infomax and the Variational Free Energy, under a single information theoretic construct, namely the view-based Information Gain. Pursuing this analytic track, two original action selection metrics named the Information Gain Lower Bound (IGLB) and the Information Gain Upper Bound (IGUB) are then proposed. Showing either a conservative or an optimistic bias regarding the Information Gain, they strongly simplify its calculation. An original fovea-based visual scene decoding setup is then proposed, with numerical experiments highlighting different facets of artificial fovea-based vision. A first and principal result is that state-of-the-art recognition rates are obtained with fovea-based saccadic exploration, using less than 10% of the original image's data. Those satisfactory results illustrate the advantage of mixing predictive control with accurate state-of-the-art predictors, namely a deep neural network. A second result is the sub-optimality of some classical action-selection metrics widely used in the literature, that is not manifest with finely-tuned inference models, but becomes patent when coarse or faulty models are used. Last, a computationally-effective predictive model is developed using the IGLB objective, with pre-processed visual scan-path read-out from memory, bypassing computationally-demanding predictive calculations. This last simplified setting is shown effective in our case, showing both a competing accuracy and a good robustness to model flaws.

## 1. Introduction

In complement with goal-oriented activity, animal motor control also relates to the search for sensory cues in order to better interpret its sensory environment and improve action efficacy. This resorts to choosing relevant viewpoints, i.e., selecting body placement and/or sensors orientation in order to capture a sensory signal that should help disambiguate the current scene. The center of sight, in particular, is constantly and actively moving during all waking time. This permanent visual scanning is principally done with high-speed targeted eye movements called saccades (Yarbus, 1967), that sequentially capture local chunks of the visual scene. This makes the oculo-motor activity an essential element of man and animal behavior, underlying most of daily displacements, movements, instrumental and social interactions.

Scene decoding through action (or “active perception”) has attracted strong interest in robotics and artificial vision, for the important redundancy present in the sensory data allows to envisage energy-efficient sensors, scanning little portions only of the total sensory scene. The opportunity to neglect large parts of the sensory scene should mainly be considered when the energy is scarce, as it is the case for drones and robots. It is also relevant in computer vision, where mega-pixel images appeals for selective convolutions, in order to avoid unnecessary matrix products. The example of animal vision thus encourages a more parsimonious approach to robotic and computer vision, *including the control of the sensory flow*. Optimizing the sensor displacements across time may then be a part of robotic control, in combination with goal-oriented operations.

Changing the viewpoint can be seen as a way to leverage ambiguities present in the current visual field. In Aloimonos et al. (1988), the authors show that some ill-posed object recognition problems become well-posed as soon as several views on the same object are considered. A more general perspective is developed in Bajcsy (1988), with a first attempt to interpret active vision in the terms of sequential Bayesian estimation:

*The problem of Active Sensing can be stated as a problem of controlling strategies applied to the data acquisition process which will depend on the current state of the data interpretation and the goal or the task of the process*.

thus providing a roadmap for the development of active sensory systems.

Work on active vision control is quite scarce until the late 2000's. On the machine learning side, an example of fovea-based visuo-motor control was addressed in Schmidhuber and Huber (1991), with a direct policy learning from gradient descent by using BPTT through a pre-processed forward model. On the biological side, early models from the late nineties consider the case of fovea-based image encoding, ending up in the simplified “pyramidal” focal image encoding model (Kortum and Geisler, 1996). Active vision models were however largely dominated by the *salience* models (Itti and Koch, 2000, 2001; Itti and Baldi, 2005), that were shown consistent with the preferred fixation zones observed in humans. Motor control were however generally bypassed in that case, putting the focus on characterizing the attractiveness of fixation zones rather that explaining the scene decoding process when changing gaze orientation.

In contrast, global scene understanding implies to consider the visual scan-path as a sequential *sampling* of an underlying (covert) sensory scene, given a generative model. Two parallel research tracks adopted and refined this last idea over the last 20 years. On the one side, a *predictive* approach to active vision was originally developed in (Najemnik and Geisler, 2005). It globally complies with the predictive coding framework (Rao and Ballard, 1999) with the current posterior estimate used to anticipate future sensations. Here, appropriate samples should be selected that maximize the expected *decoding accuracy*, that resorts to reduce the number of possible interpretations of the underlying scene, i.e., reduce the expected posterior *entropy* (see Najemnik and Geisler, 2005, 2009; Butko and Movellan, 2010; Friston et al., 2012). It also generalizes to the case of multi-view selection in object search and scene recognition (Potthast et al., 2016). A second research track insists on the formal contribution of action in the *encoding* of the (future) sensory field. This resorts to consider action as a *code* that is later on revealed (decoded) by sensing the effect of action at the visual field (Klyubin et al., 2005; Tishby and Polani, 2011). As such it may be optimized so as to maximize the action read-out capability, allowing to improve both the policy and the data model in the course of learning (Schmidhuber, 2007; Mohamed and Rezende, 2015; Houthooft et al., 2016).

Those different approaches interestingly conduct to develop different action selection *policies* that do not appear mutually compatible in the first place. The decoding accuracy objective encourages actions that provide a consistent belief update, measured at the log likelihood of the data after sampling. This implies to avoid surprising data and prefer actions that bring out a sensory input that is consistent with the initial guess (Friston, 2010). This approach may be referred as the “conservative” approach to action selection. Conversely,the “maximum effect” principle encourages actions that are well discriminated, i.e., that have a visible effect on the sensors. This is formally quantified by the “empowerment” information gain objective (Klyubin et al., 2005; Tishby and Polani, 2011), or by the more informal measures of surprise, like the “Salience” metric (Itti and Baldi, 2005), or the different “curiosity” metrics, like the ones proposed in Schmidhuber (1991), Oudeyer and Kaplan (2008), and Pathak et al. (2017). This second approach may be referred as the “progressive” approach to action selection.

Active vision is thus in need for clarification, in order to develop more effective and principle-grounded action-selection controllers in open environments. This article is an attempt to set the ground for such a unifying framework, making easier both a formal and quantitative comparisons between the different action selection metrics at stake. A fovea-based visuo-motor control setup is used for illustration, that consists in choosing the next saccade in a sequential visual scene decoding task.

A general active scene decoding framework is first developed in section 2.1, under predictive control assumptions, with a generative model explaining how the observed data is shaped by action. Stemming from a partially observed probabilistic framework, the current observation is interpreted as the realization of a *mixed emission density* made of a controlled emitter (i.e., the actuator state) and an uncontrolled one (i.e., the latent state of the environment). Then, when combined with a chain rule-based sequential update, it is shown how the (unobserved) latent state shall be inferred from both the current observation and past inferences memory. Given that “mixed” generative model, a generic active inference framework is developed in section 2.2, with classical action selection metrics recast, showing a clear formal separation between the “Accuracy-based,” “Innovation-based,” and “Conservation-based” action-selection metric families.

Section 3 gathers both formal and simulation results, providing a comprehensive and consistent interpretation of most existing metrics as maximizing the view-based Information Gain, either in an “optimistic” or in a “pessimistic” fashion. The connection between action-selection metrics and the Information Gain is first formally unfolded in section 3.1. It is shown that a rather straightforward approximation of the Information Gain, known as the Compression Improvement, provides both a general setup to interpret most classic objective functions, and a baseline to provide new effective and principle-grounded objective functions, namely the Information Gain Lower Bound (IGLB) and the Information Gain Upper Bound (IGUB). Then an actual implementation of a sequential fovea-based scene decoding setup is developed in section 3.2, allowing to quantitatively compare those different metrics, and propose new avenues toward parsimonious active vision through computationally-effective model-based prediction.

## 2. Principles and Methods

We consider here a *scene decoding task* where an agent has to estimate its environment state, here called the “sensory scene,” from sensory samples. The visual scene is organized in objects (or objects parts), whose presence and position is continuously checked by visual inspection. Then, decoding a visual scene through saccades consists in identifying the ensemble through the sequential foveation of parts of the scene only.

### 2.1. A Mixed Generative Model

The active inference approach relies on a longstanding history of probabilistic modeling in signal processing and control (Kalman, 1960; Baum and Petrie, 1966). The physical world takes the form of a random process that is the cause of the sensory stream. This process is not visible in itself but only sensed through (non reliable) sensors, providing a sequence of observations over time. The inference problem consists in identifying the cause of the observations (i.e., the state of the environment), given the generative model. The result of the inference is itself a probability density over the hidden states (the posterior probability), that is obtained through inverting the model (from the observations toward the hidden states).

#### 2.1.1. One Scene, Many Views

A feedback control framework is composed of an actor and an environment. The actor and the environment interact according to a feedback loop. The actor can act on the environment through its effectors, and sense the state of the environment through its sensors. The state of the environment as well as the state of the agent can change over time. The state of the environment is described by a state vector s∈S. The signal ***x*** that is measured on the sensors is called the *sensory field*. It is interpreted as a measure made by the sensors, that is causally related to the current state ***s***.

We consider here an organization of the environment in objects (or object parts), whose presence and position is continuously checked by sensori-motor inspection. In a (discrete) Markovian framework, the state ***s*** in which the physical system is found at present depend both on its previous state (say ***s***_0_) and on a preceding motor command ***a***. The transition from ***s***_0_ to ***s*** is reflected in a *transition* probability that embodies the deterministic and non-deterministic effects of the command ***a*** in the form of a conditional probability:

(1) $$\begin{array}{r} \text{s}\sim \text{Pr}\left(S|\text{a},{\text{s}}_{0}\right) \end{array}$$

The signal ***x*** measured on the sensors is interpreted as an effect of the current state ***s***. Once again the deterministic and non-deterministic effects are reflected in a conditional probability:

(2) $$\begin{array}{r} \text{x}\sim \text{Pr}\left(X|\text{s}\right) \end{array}$$

that is said the *sensory emission* process. The combination of (1) and (2) is the *generative process* that is the cause of the sensory field. Consider now the cause ***s*** of the current visual field ***x*** is both the object identity ***o***, its position in the peripheral space ***y***, and the current body orientation ***u***, i.e., ***s*** = (***y***, ***o***, ***u***), with ***x*** ~ Pr(*X*|***y***, ***o***, ***u***) the sensory emission. Here each variable accounts for a distinct degree of freedom responsible for the sensory emission.

Then we propose to split the generative process in two parts, namely the controlled generative process and the uncontrolled generative process. This separation is consistent with the “hidden state”/“hidden control” distinction stated in Friston et al. (2012). The controlled emitter is ***u*** while the uncontrolled emitter is (***y***, ***o***). Moreover, for greater simplicity, (***y***, ***o***) is here reduced to a single variable ***z*** = (***y***, ***o***), so that the generic uncontrolled state ***z*** may report for every possible composition of object identity in space (or more generally every composition of a pose and an identity). The controlled emitter ***u*** refers to the state of a motor apparatus, e.g., to the spatial distribution of the different mobile segments of an articulated body. The uncontrolled latent emitter **z** refers to the remaining part of the physical world, i.e., the “environment.”

This restricted setup, that separates a body and an environment in the form of two independent processes, provides a substantial simplification to the estimation problem at stake (see Appendix A in Supplementary Material). The controlled transition is assumed to be relatively “fast” in comparison with the uncontrolled one (for e.g., saccades can be realized in a 100–200 ms interval). Consistently with the “end-effector” ballistic control setup (Mussa-Ivaldi and Solla, 2004), the motor command ***a*** is thus assimilated with a setpoint (or posture) ***u*** in the actuator space. Under that perspective, the motor command acts on the sensors position and orientation so as to achieve a certain perspective (or view) over the external scene, here called a *viewpoint*.

Finally, both ***x*** (the view) and ***z*** (the *latent state*) are the realization of a generative model parametrized by ***u*** (the viewpoint), i.e.,

(3) $$\begin{array}{r} \text{x},\text{z}|\text{u}\sim \text{Pr}\left(X|Z,\text{u}\right),\text{Pr}\left(Z\right) \end{array}$$

with Pr(*Z*) the *prior*, and each different motor command ***u*** providing a different sample over the same underlying distribution. With that respect, the action ***u*** is also interpreted as a *sampling* operation.

#### 2.1.2. Sequential Bayesian Inference

With a generative model comes the possibility to *infer* the latent state of the physical system from the observation using Bayes rule:

(4) $$\begin{array}{r} \text{Pr}\left(Z|\text{x},\text{u}\right)=\frac{\text{Pr}\left(\text{x}|Z,\text{u}\right)\text{Pr}\left(Z\right)}{\text{Pr}\left(\text{x}|\text{u}\right)} \end{array}$$

with Pr(*Z*|***x***, ***u***) the *posterior* probability (over the latent states), whose order 2 moment informs on the estimation accuracy : the narrower the distribution, the more accurate the latent state prediction.

In a visual scene decoding task, a single latent state ***z*** is observed through a series of viewpoints ***u***, ***u***′, ***u***″, …This sequence of observations should ultimately provide a final estimate $\hat{q}\left(Z\right)$, with a single cause $\hat{\text{z}}$ dominating the other ones, allowing to reach a final decision. The chaining of the posterior to the role of the prior in the next inference step is a classical property of sequential Bayesian inference. When generalized to many observations: (***x***|***u***), (***x***′|***u***′), …, (***x***^(*n*)^|***u***^(*n*)^), the final posterior *q*^(*n*)^(*Z*) writes:

(5) $$\begin{array}{r} q^{\left(n\right)}\left(Z\right)\propto \text{Pr}\left(\text{x}|Z,\text{u}\right)\times \text{Pr}\left({\text{x}}^{\prime }|Z,{\text{u}}^{\prime }\right)\times \ldots \times \text{Pr}\left({\text{x}}^{\left(n\right)}|Z,{\text{u}}^{\left(n\right)}\right)\times \text{Pr}\left(Z\right) \end{array}$$

which allows to approach the latent state ***z*** from many samples of (3), each sample providing more evidence. When the sampling is done incrementally (Wald, 1945), the ***u***'s and ***x***'s do not need to be stored in the process. At step *n*, only *q*^(*n*−1)^ (the current “belief”) needs to be memorized to estimate *q*^(*n*)^, i.e.,

(6) $$\begin{array}{r} q^{\left(n\right)}\left(Z\right)\propto \text{Pr}\left({\text{x}}^{\left(n\right)}|Z,{\text{u}}^{\left(n\right)}\right)\times q^{\left(n-1\right)}\left(Z\right) \end{array}$$

### 2.2. Active Vision and Predictive Control

Consider an agent having to estimate its environment state ***z*** from sampling it from different viewpoints. We here suppose that a generative model *p* is given to the agent. Depending on the current viewpoint ***u***, a different view ***x*** is observed at the sensors. So, each different command ***u*** provokes a different observation, and thus a different estimation of the latent state. It is thus worth to question what is the optimal choice for ***u*** in order to maximize the accuracy of the posterior estimate? That turns to minimize the number of samples so as to provide an accurate estimate. This approach to inference is called *active sampling* in Friston et al. (2012), for the choice of ***u*** determines the sensory sample ***x*** that is observed, conditioning the final posterior estimate. It was originally developed by Najemnik and Geisler (2005) to the case of human visual search modeling (finding a target feature in an image, i.e., the “find Waldo” task).

A baseline sampling strategy is to choose ***u*** at random and condition the posterior estimate on this random action. More elaborate strategies consider the past observations to choose the most promising action $\hat{\text{u}}$. The knowledge about past observations being here absorbed in single posterior distribution *q*^(*n*−1)^, the problem turns out to design a *controller C* which, given a context *q*^(*n*−1)^, sets up an action $\hat{\text{u}}=C\left(q^{\left(n-1\right)}\right)$. Here the role of the controller is not to achieve a goal-oriented task, but to render the estimation of the latent state more accurate. The controller is said *perception-driven*.

The design of such a controller is not straightforward. On contrary to classical control, there is not definite setpoint z^*^ to which the controller is supposed to drive the external process (through model inversion for instance). By design, the actual latent state ***z*** is not visible as such and can not be compared to the inferred posterior. In order to estimate how good a motor command is, one needs to provide an estimate of the value-of-action (regarding scene understanding). There is currently no consensus about what a good value is regarding the scene decoding task.

A general strategy is thus to establish an *action selection metric*, taking either the form of an *objective function f* or a loss ℓ, that conveys a quantitative estimation of the action's contribution to the inference accuracy (resp. imprecision). Once the objective function established, a simple control strategy is to choose the action that maximizes the objective (resp. minimizes the loss), i.e.,:

(7) $$\hat{u}=\underset{u\in U}{\text{ argmin }}\ell (u)/\underset{u\in U}{\text{ argmax }}f(u)$$

Many such objective functions are proposed in the literature. They are generally referred as an *intrinsic* motivation (Oudeyer and Kaplan, 2008) by contrast with the *extrinsic* motivation that relates to the classical rewards in reinforcement learning (Sutton and Barto, 1998). Several such intrinsic reward candidates have been developed in recent years in the scene decoding context. Some of them are presented in the next paragraphs. The original formulas have been recast to show their formal correspondences, but also highlight some manifest differences between them.

#### 2.2.1. Accuracy-Based Action Selection

Given a generative model *p*(*X, U, Z*), like the one described in section 2.1, the predictive approach to perception-driven control (Najemnik and Geisler, 2005) relies on predicting an *accuracy* measure *A*(***x***, ***u***; *q*^(*n*−1)^) to choose action. The accuracy tells how good the model is at predicting ***z*** (here the target position) when viewing ***x*** at position ***u***, knowing *q*^(*n*−1)^ (the estimated posterior at step *n* − 1).

If the agent has to choose an action u∈U, knowing only *q*^(*n*−1)^, the *predicted* accuracy attached to ***u*** is:

$$\begin{array}{l} \bar{A}\left(u;q^{\left(n-1\right)}\right)=E_{z\sim q^{\left(n-1\right)}(Z),x\sim p\left(X|z,u\right)}\left[A\left(x,u;q^{\left(n-1\right)}\right)\right]\\ \;=\sum_{z\in Z}q^{\left(n-1\right)}(z)\int_{X}A\left(x,u;q^{\left(n-1\right)}\right)p\left(x|z,u\right)dx \end{array}$$

and the optimal action to choose is:

(8) $$\begin{array}{l} \hat{u}=\underset{u\in U}{\text{ argmax }}\bar{A}\left(u;q^{\left(n-1\right)}\right) \end{array}$$

In order to render the computation tractable, a sample is generally used to estimate the predicted accuracy, i.e., ${\text{E}}_{p}\left[f\left(\text{x}\right)\right]≃f\left(\tilde{\text{x}}\right)$, with $\tilde{\text{x}}\sim p\left(\text{x}\right)$.

The accuracy metric used in the original paper was an *ad-hoc* one (Najemnik and Geisler, 2005), but turned out to be consistent with minimizing the *posterior entropy* (Najemnik and Geisler, 2009), i.e.,:

$$A\left(x,u;q^{\left(n-1\right)}\right)=-H\left(q^{\left(n\right)}\right)=\sum_{z\in Z}q^{\left(n\right)}(z)\log q^{\left(n\right)}(z)$$

with: *q*^(*n*)^(***z***) ∝ *p*(***x|z***, ***u***) × *q*^(*n*−1)^(***z***), so that:

$$\hat{u}=\underset{u\in U}{\text{ argmin }}E_{z\sim q^{\left(n-1\right)}(Z),x\sim p\left(X|z,u\right)}\left[H\left(q^{\left(n\right)}\right)\right]$$

which makes sense for a low entropy of the posterior is expected when the estimated posterior accuracy is high.

This approach to optimal visual sampling was further on linked to an “Infomax” principle(posterior Mutual Information maximization) in Butko and Movellan (2010), taking:

(9) $$A_{\text{INFOMAX}}(x,u;q^{\left(n-1\right)})\equiv I(Z;x|u;q^{\left(n-1\right)})=H(q^{\left(n-1\right)})-H(q^{\left(n\right)})$$

with *H*(*q*^(*n*−1)^) ≡ *H*(*Z*|*q*^(*n*−1)^) and *H*(*q*^(*n*)^) ≡ *H*(*Z*|***x***, ***u***; *q*^(*n*−1)^), which also turns out to minimize *H*(*q*^(*n*)^) solely for *H*(*q*^(*n*−1)^) is independent of ***u***. The Infomax (or posterior entropy minimization) approach generally makes sense for it implicitly relies on the chaining from *q*^(*n*−1)^ to *q*^(*n*)^, that considers that if *p*(***x***|*Z*, ***u***) is consistent with *q*^(*n*−1)^(*Z*), then the issued posterior entropy should be lower than if *p*(***x***|*Z*, ***u***) is at odd with *q*^(*n*−1)^(*Z*). The model is expected to choose the action that may confirm the initial assumption, though there is no formal comparison between *q*^(*n*−1)^ and *q*^(*n*)^. It is thus potentially vulnerable to model outliers with *q*^(*n*)^ having both a low entropy and being inconsistent with *q*^(*n*−1)^.

#### 2.2.2. Innovation-Based Action Selection

Another quantity of interest is the so-called *Bayesian surprise* or *Salience* (Itti and Baldi, 2005) defined as the Kullback-Leibler divergence between an actual view ***x*** and a model ***z***. In the original “bottom-up” setup, only local statistics are formed over small image patches of a given image, with ***u*** the index of a patch and *p*(***z***|***x***, ***u***) the features inferred from the data actually observed at ***u***. For each patch ***u***, the Salience of the actual view ***x*** given the model is:

S(x,u)=KL(p(Z|x,u)||p(Z))

with $\text{KL}\left(p_{1}||p_{2}\right)=\underset{z}{\sum }p_{1}\left(\text{z}\right)\log\frac{p_{1}\left(\text{z}\right)}{p_{2}\left(\text{z}\right)}$ the Kullback-Leibler divergence between *p*_1_ and *p*_2_, interpreted here as a measure of the *in*consistency between a (viewpoint independent) model *p* and the data. A high salience reflects a strong inconsistency with the model, while a low salience reflects a strong consistency with the model. According to Itti and Baldi, the regions that have a high Bayesian surprise are the ones that attract the sight the most. The calculation of *S*(***x***, ***u***) at each location ***u*** forms a *saliency map* that is then considered as a prediction of where the sight will most likely be attracted (high values most probably attract the sight, low values less probably do). The saliency model has a strong explanatory power and provides among the best fit with the actual preferred fixation zones observed in humans. Its scalability moreover provides straightforward applications in image and video compression (Wang et al., 2003; Guo and Zhang, 2010).

Generalized to the sequential setup, the saliency metric becomes:

(10) $$\begin{array}{l} S(x,u;q^{\left(n-1\right)})=\text{KL}(q^{\left(n\right)}||q^{\left(n-1\right)}) \end{array}$$

with *q*^(*n*−1)^ considered as the data model and *q*^(*n*)^ the posterior estimated at (***x***, ***u***), knowing *q*^(*n*−1)^. Through maximizing the KL divergence between the previous and the current scene interpretation, the Saliency objective is found here to promote the most *conflicting* observation regarding previous assumptions, entailing finding innovative interpretations of the current scene.

Put in a predictive form, it gives:

$$\hat{u}=\underset{u\in U}{\text{ argmax }}E_{z\sim q^{\left(n-1\right)},x\sim p(X|z,u)}\left[\text{KL}(q^{\left(n\right)}||q^{\left(n-1\right)})\right]$$

with the predictive Saliency promoting alternate future interpretations regarding the current interpretation. This entails searching for model inconsistencies or model contradicting predictions, making the Saliency a model consistency check metric.

#### 2.2.3. Conservation-Based Action Selection

At last, the Variational Free Energy based (VFE) active inference setup (Friston, 2010; Friston et al., 2012) considers the general tendency of the brain to counteract surprising and unpredictable sensory events through minimizing the VFE with action (see Appendix B in Supplementary Material). In our sequential setup, it writes:

(11) F(x|u)=−logp(x|u)+KL(q(Z)||p(Z|x,u))

From the predictive perspective, stemming from *q*^(*n*−1)^ as the current scene interpretation, Friston et al. (2017) propose a *predictive VFE* that writes in our sequential setup like :

(12) $$\begin{array}{ll} \bar{F}(u;q^{\left(n-1\right)}) & =E_{z\sim q^{\left(n-1\right)}(Z),x\sim p(X|z,u)}\\ & \;\left[-\log p(x|u;q^{\left(n-1\right)})+\text{KL}(q^{\left(n-1\right)}||q^{\left(n\right)})\right] \end{array}$$

with *q*^(*n*)^ the predicted posterior and KL(*q*^(*n*−1)^||*q*^(*n*)^) quantifying the scene interpretation update made by interpreting the scene with *q*^(*n*)^ instead of *q*^(*n*−1)^. It is said the “epistemic cost”^1^. In that setup, minimizing the Free-Energy is consistent with minimizing KL(*q*^(*n*−1)^||*q*^(*n*)^) estimated as:

$$\text{KL}(q^{\left(n-1\right)}||q^{\left(n\right)})=E_{z\sim q^{\left(n-1\right)}(Z)}\left[\log q^{\left(n-1\right)}(z)-\log q^{\left(n\right)}(z)\right]$$

Put in a predictive form, the selection of action finally relies on reducing the predicted log ratio, i.e., :

(13) $$\hat{u}=\underset{u\in U}{\text{argmin }}E_{z\sim q^{\left(n-1\right)}(Z),x\sim p(X|z,u)}\left[\log q^{\left(n-1\right)}(z)-\log q^{\left(n\right)}(z)\right]$$

which may minimize the *epistemic cost*^2^.

On contrary to the Infomax objective (section 2.2.1), minimizing the epistemic cost selects the predicted posterior having the highest consistency with the current posterior, which may prevent from model inconsistencies that may incidently “hack” the posterior entropy (see section 2.2.1). Minimizing KL(*q*^(*n*−1)^||*q*^(*n*)^) thus corresponds to a *conservative* approach to the scene interpretation that is *minimally* vulnerable to outliers, i.e., that minimizes the risk of a conflicting interpretation.

The epistemic cost is moreover at odd with the Saliency objective (section 2.2.2) seeking the maximal *in*consistency between the cumulated posterior and the current posterior. It is obvious here that the Free Energy minimization and the Saliency maximization are antithetic objectives, and no consensus is currently observed in the literature about which objective should prevail (though Infomax generally preferred in scene decoding and saliency/surprise preferred in sparse reinforcement learning).

## 3. Results

### 3.1. View-Based Information Gain Metrics

The structure of the problem (many views on the same scene) implies that different observations should share a common information corresponding to the actual (covered) sensory scene. We take here benefit of the viewpoint-based variational encoding setup (seen in section 2.2.3 and Appendix B in Supplementary Material) to propose a new quantification the *mutual information* shared across different sensory fields, locally estimated with a view-based Information Gain metric. It is shown here that a rather straightforward approximation of the information gain, known as the Compression Improvement, provide both a general setup to interpret most classic objective functions, and a baseline to provide new effective and principle-grounded objective functions.

#### 3.1.1. Definitions

##### 3.1.1.1. View-based mutual information and information gain

The sharing of information between two sensory fields ***x***|***u*** and ***x***′|***u***′ should be quantified by their *Mutual Information*. The general idea is that two samples may provide more insight about the hidden sensory scene than a single one (three samples should provide even more, etc.). Consider ***x***|***u*** as the initial sensory sample and ***x***′|***u***′ as an additional sample providing new evidence about how interpret the initial view ***x***. The view-based mutual information writes:

(14) $$I((X|u);(X^{\prime }|u^{\prime }))=H(X|u)-H(X|u,X^{\prime },u^{\prime })$$

(15) $$≃\;E_{X,X^{\prime }}[-\log p(X|u)\text{​ }+\text{ ​}\log p(X|u,X^{\prime },u^{\prime })]$$

with :

(*i*) $p\left(\text{x}|{\text{u}}^{\prime },{\text{x}}^{\prime },\text{u}\right)≜\underset{\text{z}}{\sum }p\left(\text{x}|\text{z},\text{u}\right)p\left(\text{z}|{\text{x}}^{\prime },{\text{u}}^{\prime }\right)$ the *post-sample* likelihood, i.e., the retrospective likelihood of having seen ***x*** at ***u*** knowing now that ***x***′ is observed at ***u***′, and
(*ii*) −log*p*(***x***|***u***)+log*p*(***x***|***u***′, ***x***′, ***u***) the post-sample *Information Gain* (see also Tishby and Polani (2011)), that is a local estimator of the views mutual information at *X* = ***x*** and *X*′ = ***x***′, given the model *p*:
  (16) $$\text{IG}(x,u,x^{\prime },u^{\prime })=-\log p(x|u)+\log p(x|u^{\prime },x^{\prime },u)$$

##### 3.1.1.2. Conditional reconstruction cost

Stemming from the sequential Bayes posterior update formula:

(17) $$\begin{array}{l} p(z|x,u,x^{\prime },u^{\prime })=\frac{p(x|z,u)p(z|x^{\prime },u^{\prime })}{p(x|u,x^{\prime },u^{\prime })} \end{array}$$

It can be shown that the negative log likelihood of ***x*** after seeing both ***x*** and ***x***′ is bounded from above by:

(18) $$\begin{array}{l} -\log p(x|u,x^{\prime },u^{\prime })\leq -\sum_{z}q^{\prime }(z)\log p(x|z,u)\frac{p(z|x^{\prime },u^{\prime })}{q^{\prime }(z)}\\ \;=E_{z\sim q^{\prime }}\left[-\log p(x|z,u)\right]+\text{KL}(q^{\prime }(Z)||p(Z|x^{\prime },u^{\prime })) \end{array}$$

(19) $$\begin{array}{l} =-\log p(x|u,x^{\prime },u^{\prime })+\text{KL}(q^{\prime }(Z)||p(Z|x,u,x^{\prime },u^{\prime }))\\ ≜F(x|u,x^{\prime },u^{\prime }) \end{array}$$

which establishes *F*(***x***|***u***, ***x***′, ***u***′) as the post-sample *conditional reconstruction cost* (or *conditional Free Energy* – the two are synonyms), with *q*′(***z***) expectedly approaching *p*(***z***|***x***, ***u***, ***x***′, ***u***′) after optimization. From a variational perspective, the passing from *q*(*Z*) ≃ *p*(*Z*|***x***, ***u***) to *q*′(*Z*) ≃ *p*(*Z*|***x***, ***u***, ***x***′, ***u***′) is the *variational posterior update*, and the passing from *F*(***x***|***u***) toward *F*(***x***|***u***′, ***x***′, ***u***) is the *reconstruction cost update*.

##### 3.1.1.3. Compression improvement

An approximation of the Information Gain (IG), known as the *Compression Improvement* (CI) was proposed in Schmidhuber (2007) and Houthooft et al. (2016). In our view-based setup, it writes :

(20) $$\begin{array}{l} \text{CI}(x,u,x^{\prime },u^{\prime })=F(x|u)-F(x|u,x^{\prime },u^{\prime }) \end{array}$$

There comes the possibility to optimize the *next* sampling ***u***′ through maximizing the CI as a proxy for the IG. It happens to be equivalent with minimizing the post-sample Free Energy, consistently with Friston et al. (2012)'s intuition.

#### 3.1.2. The Sequential Information Gain and Its Approximations

Extending now to the sequential setup, the contribution of ***u***^(*n*)^ in understanding the scene is measured by a change in the reconstruction cost *F before* and *after* reading ***x***^(*n*)^|***u***^(*n*)^.

- Before reading ***x***^(*n*)^, the reconstruction cost at ***x***^(*n*−1)^ writes:
  (21) $$\begin{array}{l} F(x^{\left(n-1\right)}|u^{\left(n-1\right)};q^{\left(n-2\right)})=E_{z\sim q}\left[-\log p(x^{\left(n-1\right)}|z,u^{\left(n-1\right)})\right]\\ \;+\text{KL}(q||q^{\left(n-2\right)}) \end{array}$$
  (22) $$\begin{array}{l} =-\log p(x^{\left(n-1\right)}|u^{\left(n-1\right)};q^{\left(n-2\right)})\\ \;+\text{KL}(q||q^{\left(n-1\right)}) \end{array}$$
- After reading ***x***^(*n*)^, it writes:
  (23) $$\begin{array}{l} F(x^{\left(n-1\right)}|u^{\left(n-1\right)},x^{\left(n\right)},u^{\left(n\right)};q^{\left(n-2\right)})=E_{z\sim q^{\prime }}\left[-\log p(x^{\left(n-1\right)}|z,u^{\left(n-1\right)})\right]\\ \;+\text{KL}(q^{\prime }||q^{\left(n;n-2\right)}) \end{array}$$
  (24) $$\begin{array}{l} =-\log p(x^{\left(n-1\right)}|u^{\left(n-1\right)};q^{\left(n;n-2\right)})\;\\ \;+\text{KL}(q^{\prime }||q^{\left(n\right)}) \end{array}$$
  with :
  (25) $$\begin{array}{l} q^{\left(n;n-2\right)}(Z)\propto p(Z|x^{\left(n\right)},u^{\left(n\right)})q^{\left(n-2\right)}(Z) \end{array}$$

Before reading ***x***^(*n*)^, the optimal reconstruction cost is attained at *q* = *q*^(*n*−1)^. After reading ***x***^(*n*)^, the optimal reconstruction cost is attained at *q*′ = *q*^(*n*)^. From subtracting (23) from (21), the CI writes :

(26) $$\begin{array}{ll} {\text{CI}}^{\left(n\right)}= & E_{z\sim q}\left[-\log p(x^{\left(n-1\right)}|z,u^{\left(n-1\right)})\right]+\text{KL}(q||q^{\left(n-2\right)})\\ & +E_{z\sim q^{\prime }}\left[\log p(x^{\left(n-1\right)}|z,u^{\left(n-1\right)})\right]-\text{KL}(q^{\prime }||q^{\left(n;n-2\right)}) \end{array}$$

Knowing that *q* and *q*′ are free parameters, taking *q* = *q*′ provides a strong simplification of the above formula, further on referred as the *approximate CI*:

(27) $${\tilde{\text{C}}\text{I}}^{\left(n\right)}=\text{KL}(q||q^{\left(n-2\right)})-\text{KL}(q||q^{\left(n;n-2\right)})$$

(28) $$=E_{z\sim q}\left[\log p(z|x^{\left(n\right)},u^{\left(n\right)})\right]+c$$

with *c* a constant. The information gain is here approached with the opposite of the cross-entropy cost ${\tilde{\text{C}}\text{I}}^{\left(n\right)}=-H\left(q\left(Z\right),p\left(Z|{\text{x}}^{\left(n\right)},{\text{u}}^{\left(n\right)}\right)\right)+c$.

##### 3.1.2.1. Information gain lower bound (IGLB)

Maximizing the CI however provides no formal guarantee the IG will be maximized. The reconstruction cost is indeed an upper bound of the negative log evidence, but the difference of two reconstruction costs is neither an upper bound or a lower bound of the IG (so that it may either underestimate or overestimate the IG). It happens that, contrarily to the original CI formula (Equation 26), the approximate CI (Equation 28) provides ways to establish firm bounds regarding the Information Gain estimate. Taking for instance *q* = *q*^(*n*−1)^ (the pre-sample posterior), and subtracting (24) from (22), the approximate CI writes:

(29) $$\begin{array}{ll} {\tilde{\text{C}}\text{I}}_{q=q^{\left(n-1\right)}}^{\left(n\right)} & =-\log p(x^{\left(n-1\right)}|u^{\left(n-1\right)};q^{\left(n-2\right)})\\ & \;+\log p(x^{\left(n-1\right)}|u^{\left(n-1\right)};q^{\left(n;n-2\right)})\\ & \;-\text{KL}(q^{\left(n-1\right)}||q^{\left(n\right)}) \end{array}$$

making the approximate CI objective a *lower bound* of the IG (Equation 16), for it *underestimates* the IG by an amount equal to KL(*q*^(*n*−1)^||*q*^(*n*)^), thus providing a principled objective to optimize action, with a simple objective function defined here as the *Information Gain Lower Bound* (IGLB):

(30) $$\begin{array}{ll} {\tilde{\text{C}}\text{I}}_{q=q^{\left(n-1\right)}}^{\left(n\right)} & ≜\text{IGLB}(x^{\left(n\right)},u^{\left(n\right)},q^{\left(n-1\right)})\\ & \;=E_{z\sim q^{\left(n-1\right)}}\left[\log p(z|x^{\left(n\right)},u^{\left(n\right)})\right]+c \end{array}$$

It is maximal when *p*(*Z*|***x***^(*n*)^, ***u***^(*n*)^) ≃ *q*^(*n*−1)^(*Z*), i.e., when the current posterior *p*(*Z*|***x***^(*n*)^, ***u***^(*n*)^) and the past cumulated posterior *q*^(*n*−1)^ are highly consistent.

If now ***u*** is at choice *before* sampling ***x***, it is sensible to maximize the predicted IGLB to maximize the code consistency, i.e.,:

(31) $$\begin{array}{ll} \hat{u} & =\underset{u\in U}{\text{ argmax }}E_{z\sim q^{\left(n-1\right)};x\sim p(X|z,u))}\left[\log p(z|x,u)\right] \end{array}$$

The amount of the underestimation, i.e., KL(*q*^(*n*−1)^||*q*^(*n*)^) defines the epistemic cost (see section 2.2.3) as the *IGLB bias*, that should be minimized through action (see Equation 12). Minimizing this term means reducing the underestimation made about the future information gain *before posterior update*. This means searching for “accurate” Information Gains approximations, rather than maximizing the Information Gain itself, that links the IGLB with “safe” or “conservative” action selection policies.

At last, the IGLB objective containing both the IG objective and the (negative) epistemic cost, maximizing the IGLB is expected to both promote high information gains and prevent for conflicting predictions, making it a “conservative” information-seeking objective.

##### 3.1.2.2. Information gain upper bound (IGUB)

For completeness, instantiating *q* with *q*^(*n*)^ gives a different objective that writes:

(32) $$\begin{array}{ll} {\tilde{\text{C}}\text{I}}_{q=q^{\left(n\right)}}^{\left(n\right)} & =-\log p(x^{\left(n-1\right)}|u^{\left(n-1\right)};q^{\left(n-2\right)})\\ & \;+\log p(x^{\left(n-1\right)}|u^{\left(n-1\right)};q^{\left(n;n-2\right)})+\text{KL}(q^{\left(n\right)}||q^{\left(n-1\right)}) \end{array}$$

making it an upper bound of the IG that *overestimates* the information gain by an amount equal to KL(*q*^(*n*)^||*q*^(*n*−1)^).

If now ***u*** is at choice *before* sampling ***x***, maximizing the approximate CI gives:

(33) $$\begin{array}{l} \hat{u}=\underset{u\in U}{\text{ argmax }}E_{z\sim q^{\left(n-1\right)};x\sim p(X|z,u))}\left[E_{z^{\prime }\sim q^{\left(n\right)}}\log p(z^{\prime }|x,u)\right]\\ \;=\underset{u\in U}{\text{ argmax }}E_{z\sim q^{\left(n-1\right)};x\sim p(X|z,u))}\left[-H(q^{\left(n\right)}(Z),p(Z|x,u))\right]\\ \;=\underset{u\in U}{\text{ argmax }}E_{z\sim q^{\left(n-1\right)};x\sim p(X|z,u))} \end{array}$$

(34) $$\left[-H(q^{\left(n\right)})-\text{KL}(q^{\left(n\right)}(Z)||p(Z|x,u))\right]$$

that interestingly combines the Infomax objective (Equation 9) with a consistency objective. This mixed objective is further on referred as the *Information Gain Upper Bound* (IGUB):

(35) $$\begin{array}{l} \text{IGUB}(x,u,q^{\left(n-1\right)})≜-H(q^{\left(n\right)})-\text{KL}(q^{\left(n\right)}(Z)||p(Z|x,u))+c \end{array}$$

The IGUB bias, i.e., KL(*q*^(*n*)^||*q*^(*n*−1)^), interestingly fits the Saliency objective (Equation 10), allowing to interpret the Saliency as the amount of the *overestimation* made *after posterior update* by considering (28) instead of (26).

Maximizing the Saliency is here interpreted as promoting the most optimistically biased Information Gain approximation, irrespectively of the Information Gain itself, making it a “risk-seeking” (rather than information seeking) objective, that links with exploratory behavior. At last, the IGUB objective containing both the IG objective and the Saliency, maximizing the IGUB is expected to both promote high information gains and promote conflicting predictions, making it an “optimistic” information-seeking objective.

To conclude, remarkable here is the fact that both the Saliency objective, the Infomax objective and the Free Energy epistemic cost show a consistent inclusion in a more general approximate Information Gain maximization principle. The final set of metrics to be compared in next section are finally displayed in Table 1.

**Table 1:** Action selection metrics summary.

**Name**	**Value**	**Equation #**	**Interpretation**
Infomax	*H*(*q*^(*n*−1)^)−*H*(*q*^(*n*)^)	(9)	Posterior mutual information maximization
Saliency	KL(*q*^(*n*)^||*q*^(*n*−1)^)	(10)	Posterior *in*consistency maximization
VFE	−log*p*(***x***^(*n*)^|***u***^(*n*)^; *q*^(*n*−1)^)+KL(*q*^(*n*−1)^||*q*^(*n*)^)	(11)	Prior *in*consistency minimization
Compression Improvement	$E_{\text{z}\sim q^{\left(n-1\right)}}\left[-\log p\left({\text{x}}^{\left(n-1\right)}|\text{z},{\text{u}}^{\left(n-1\right)}\right)\right]+\text{KL}\left(q^{\left(n-1\right)}||q^{\left(n-2\right)}\right)$	(26)	(approximate) Information Gain maximization
	$+E_{\text{z}\sim q^{\left(n\right)}}\left[\log p\left({\text{x}}^{\left(n-1\right)}|\text{z},{\text{u}}^{\left(n-1\right)}\right)\right]-\text{KL}\left(q^{\left(n\right)}||q^{\left(n;n-2\right)}\right)$		
IGLB	$E_{z\sim q^{\left(n-1\right)}}\left[\log p\left(\text{z}|{\text{x}}^{\left(n\right)},{\text{u}}^{\left(n\right)}\right)\right]+c$	(30)	(pessimistic) Information Gain maximization
IGUB	$E_{z\sim q^{\left(n\right)}}\left[\log p\left(\text{z}|{\text{x}}^{\left(n\right)},{\text{u}}^{\left(n\right)}\right)\right]+c$	(35)	(optimistic) Information Gain maximization

### 3.2. Fovea-Based Visual Scene Decoding

We now turn to an actual implementation of a saccadic visual scene decoding setup. The image (i.e., the visual scene to decode) is analyzed through a finite sequence of local *foveated* visual samples. The control problem consists in choosing the next saccade, given the past observations and the current scene interpretation. The final decoding means identifying to which category the image belongs to (here the label of the MNIST digits dataset). In that setup,

- The viewpoint ***u*** is defined as the current gaze orientation on the image (i.e., the central fixation setpoint in pixel coordinates),
- The view ***x***|***u*** is a retinocentric visual sample measured at position ***u***, with central magnification and peripheral blurring,
- The latent state ***z*** (visual scene interpretation) is the category of the image (here a digit label), to be guessed from several visual samples.

#### 3.2.1. Fovea-Based Vision

##### 3.2.1.1. Pyramidal fovea-based visual observation

In superior vertebrates, two principal tricks are used to minimize sensory resource consumption in scene exploration. The first trick is the foveated retina, that concentrates the photoreceptors at the center of the retina, with a more scarce distribution at the periphery (Osterberg, 1935). A foveated retina allows both treating central high spatial frequencies, and peripheral low spatial frequencies at a single glance (i.e., process several scales in parallel). The second trick is the sequential saccadic scene exploration, already mentioned, that allows to grab high spatial frequency information where it is necessary (serial processing).

**Figure 1:**
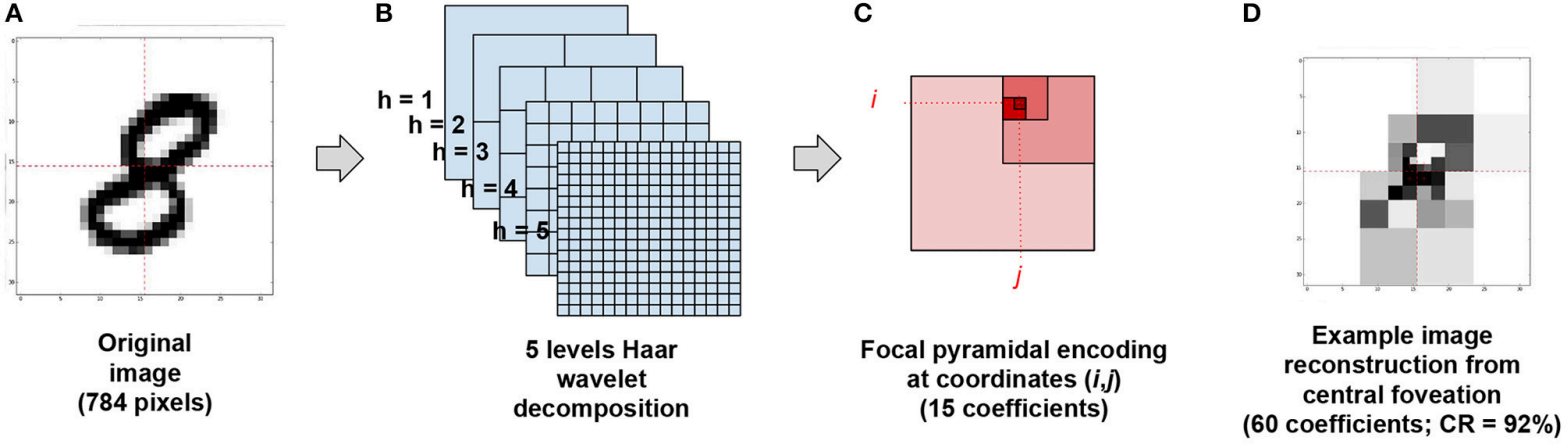
**A–C** Foveal “pyramidal” encoding from image. **(A)** An original MNIST sample is recast on a 32 × 32 grid. **(B)** It is then decomposed in a five levels Haar wavelets decomposition issuing a total of 1024 wavelet coefficient. **(C)** Then, for each gaze orientation (*i, j*)∈{0, …, 15}^2^, 3 × 5 wavelet coefficients are read out at coordinates (*i, j*), $\left(\left\lfloor \frac{i}{2}\right\rfloor ,\left\lfloor \frac{j}{2}\right\rfloor \right)$, $\left(\left\lfloor \frac{i}{4}\right\rfloor ,\left\lfloor \frac{j}{4}\right\rfloor \right)$, $\left(\left\lfloor \frac{i}{8}\right\rfloor ,\left\lfloor \frac{j}{8}\right\rfloor \right)$ and $\left(\left\lfloor \frac{i}{16}\right\rfloor ,\left\lfloor \frac{j}{16}\right\rfloor \right)$ in level descending order. **(D)** Example image reconstruction from reading 60 central coefficients at coordinates (7,7), (7,8), (8,7) and (8,8), issuing a 92% compression rate.

The baseline vision model we propose relies first on learning local foveated views on images. Consistently with (Kortum and Geisler, 1996; Wang et al., 2003), we restrain here the foveal transformation to its core algorithmic elements, i.e., the local compression of an image according to a particular focus. Our foveal image compression thus rests on a “pyramid” of 2D Haar wavelet coefficients placed at the center of sight. Taking the example of the MNIST dataset^3^ (see Figure 1A), we first transform the original images according to a 5-levels wavelet decomposition (see Figure 1B). We then define a viewpoint ***u*** = (*i, j, h*) as a set of 3 coordinates, with *i* the row index, *j* the column index and *h* the spatial scale. Each ***u*** generates a visual field made of three wavelet coefficients ${\text{x}}_{i,j,h}≜\text{x}|\left(i,j,h\right)\in {\mathbb{R}}^{3}$, obtained from an horizontal, a vertical and an oblique filter at location (*i, j*) and scale *h*. The multiscale visual information ${\text{x}}_{i,j}≜\text{x}|\left(i,j\right)\in {\mathbb{R}}^{15}$ available at coordinates (*i, j*) corresponds to a set of 5 coefficient triplets, namely:

(36) $$\begin{array}{l} x_{i,j}=\left\{x_{i,j,5},x_{\left\lfloor i/2\right\rfloor ,\left\lfloor j/2\right\rfloor ,4},x_{\left\lfloor i/4\right\rfloor ,\left\lfloor j/4\right\rfloor ,3},x_{\left\lfloor i/8\right\rfloor ,\left\lfloor j/8\right\rfloor ,2},x_{\left\lfloor i/16\right\rfloor ,\left\lfloor j/16\right\rfloor ,1}\right\} \end{array}$$

(see Figure 1C), so that each multiscale visual field owns 15 coefficients only (to be compared with the 784 pixels of the original image). Figure 1D displays a reconstructed image from the 4 central viewpoints at coordinates (7, 7), (7, 8) (8, 7) and (8, 8).

##### 3.2.1.2. Algorithms

A generic sequential scene decoding setup is provided in algorithms 1 and 2. A significant algorithmic add-on when compared with formula (8) is the use of a *dynamic actions set* : U. At each turn, the new selected action ũ is drawn off from U, so that the next choice is made over fresh directions that have not yet been explored. This implements the inhibition of return principle stated in Itti and Koch (2001). A second algorithmic add-on is the use of a threshold *H*_ref_ to stop the evidence accumulation process when enough evidence has been gathered. This threshold is a free parameter of the algorithm that sets whether we privilege a conservative (tight) or optimistic (loose) threshold. The stopping criterion needs to be optimized to arbitrate between resource saving and decoding accuracy.

**Algorithm 1:** Prediction-Based Policy

**Require:** *p* (emission density), ρ (prior), *A* (objective function), U (actions set)
**for** z,u∈Z,U **do**
*predict:* ${\tilde{\text{x}}}_{z,u}\sim p\left(X|\text{z},\text{u}\right)$
$r\left(\text{z},\text{u}\right)\leftarrow A\left(\text{z},\text{u},{\tilde{\text{x}}}_{z,u},p,\rho \right)$
**end for**
**return** $\tilde{\text{u}}=\underset{\text{u}\in U}{\text{argmax}}\langle \rho ,r\left(:,\text{u}\right)\rangle$

**Algorithm 2:** Scene Exploration

**Require:** *p* (emission density), ρ_0_ (initial prior), *A* (objective), U (actions set)
ρ←ρ_0_
**while** *H*(ρ)>*H*_ref_ **do**
*choose:* $\tilde{\text{u}}\leftarrow \text{Prediction-Based Policy}\left(p,\rho ,A,U\right)$
*read:* ***x***_*ũ*_
*update:* $\text{odd}\leftarrow \log p\left({\text{x}}_{ũ}|Z,\tilde{\text{u}}\right)+\log\rho$
ρ←softmax(odd) {*the posterior becomes the prior of the* *next turn*}
$U\leftarrow U\backslash \left\{\tilde{\text{u}}\right\}$
**end while**

The actual saccade exploration algorithm moreover adapts algorithm 2 the following way. The process starts from a loose assumption based on reading the root wavelet coefficient of the image, from which an initial guess ρ_0_ is formed. Then, each follow-up saccade is defined as the gaze end-orientation (*i, j*)∈[0, .., 15]^2^. The posterior calculation rests on up to 5 coefficient triplets (see Equation 36). After selecting gaze orientation (*i, j*), all the corresponding coordinates (*i, j, h*)'*s* are discarded from U and can not be reused for upcoming posterior estimation (for the final posterior estimate may be consistent with a uniform scan over the wavelet coefficients).

##### 3.2.1.3. Baseline generative model

A generative model is learned for each ***u*** = (*i, j, h*) (making a total of 266 data models) over the 55,000 examples of the MNIST training set. For each category ***z*** and each viewpoint ***u***, a generative emission model is built over parameter set Θ_***z***, ***u***_ = (ρ_***z***, ***u***_, ***μ***_***z***, ***u***_, **Σ**_***z***, ***u***_),

so that:

(37) $$\begin{array}{l} \forall z,u,{\tilde{x}}_{z,u}\sim p(X|z,u)=ℬ(\rho_{z,u})\times N(\mu_{z,u},\Sigma_{z,u}) \end{array}$$

with B a Bernouilli distribution and N a multivariate Gaussian. The role of the Bernouilli is to “gate” the multivariate Gaussian model in the high frequencies, where digit deformations is reflected in an alternating presence or absence of pixels for high level coefficients and at the periphery, allowing to discard the “white” triplets from the Gaussian moments calculation. Each resulting emission density *p*(*X*|*Z*, ***u***) is a mixture of Bernouilli-gated Gaussians over the 10 MNIST labels. On the inference side, the posterior is explicitly calculated using Bayes rule, i.e., *q*(*Z*|***x***, ***u***) = softmax log *p*(***x***|*Z*, ***u***), issuing about 92% recognition rate on the MNIST test set when combining the 266 log likelihoods of each wavelet triplet of the full images with Equation (5), a typical recognition rate for shallow models.

#### 3.2.2. Metrics Comparison

##### 3.2.2.1. Baseline model and decoding compression

**Figure 2:**
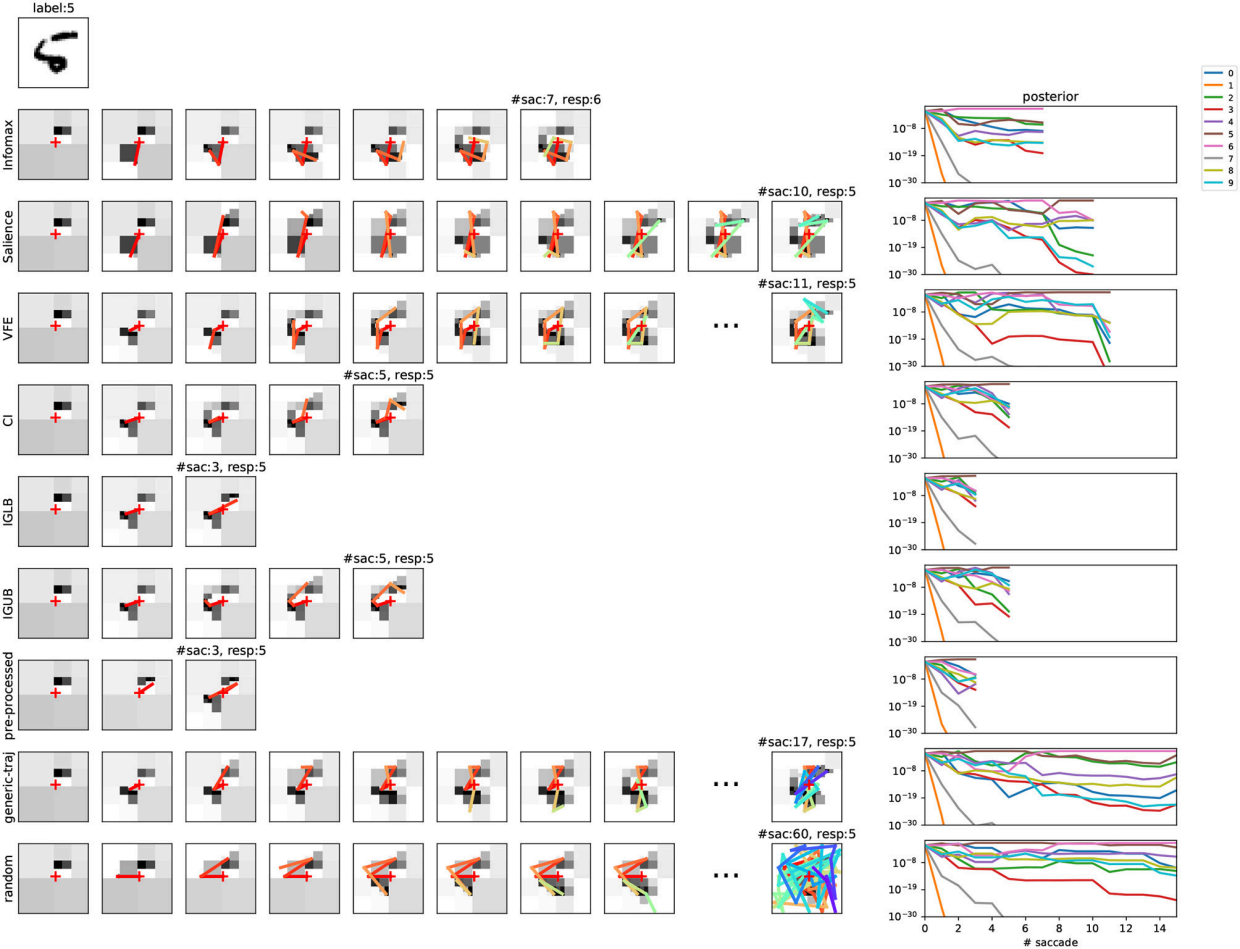
Scene exploration through saccades in the foveated vision model. **(Top left)** Original MNIST sample to be decoded, with corresponding label. **(Left panel)** Course of saccades for different action selection metrics. Leftmost is the metric name. For each row, the number of thumbnail images reflects the number of saccades. The scene decoding reads from left to right: more wavelet coefficients are grabbed at each step, visually reflected in an increased reconstruction neatness. On overlay is the corresponding history of visual fixations (with rainbow time color code). The total number of saccades can vary for the different policies. Over the final thumbnail is the number of saccades and the final response. All decoding steps are shown except when *n*>10. **(Right panel)** corresponding posterior update in function of the number of decoding steps, for 0 ≤ *n* ≤ 15 (y-logarithmic scale, one color per competing label). Baseline model (Equation 37); $H_{\text{ref}}=10^{-4}$.

Different examples of a sequential scene decoding are presented in Figure 2 for one MNIST sample using algorithm 2 and different objective functions. Note that several coefficient triplets are read at each end-effector position (*i, j*) (see Figure 1C). There is for instance a total of 5 triplets read out at the initial gaze orientation, and between 1 and 4 triplets read-out for each continuing saccade (not shown).

**Figure 3:**
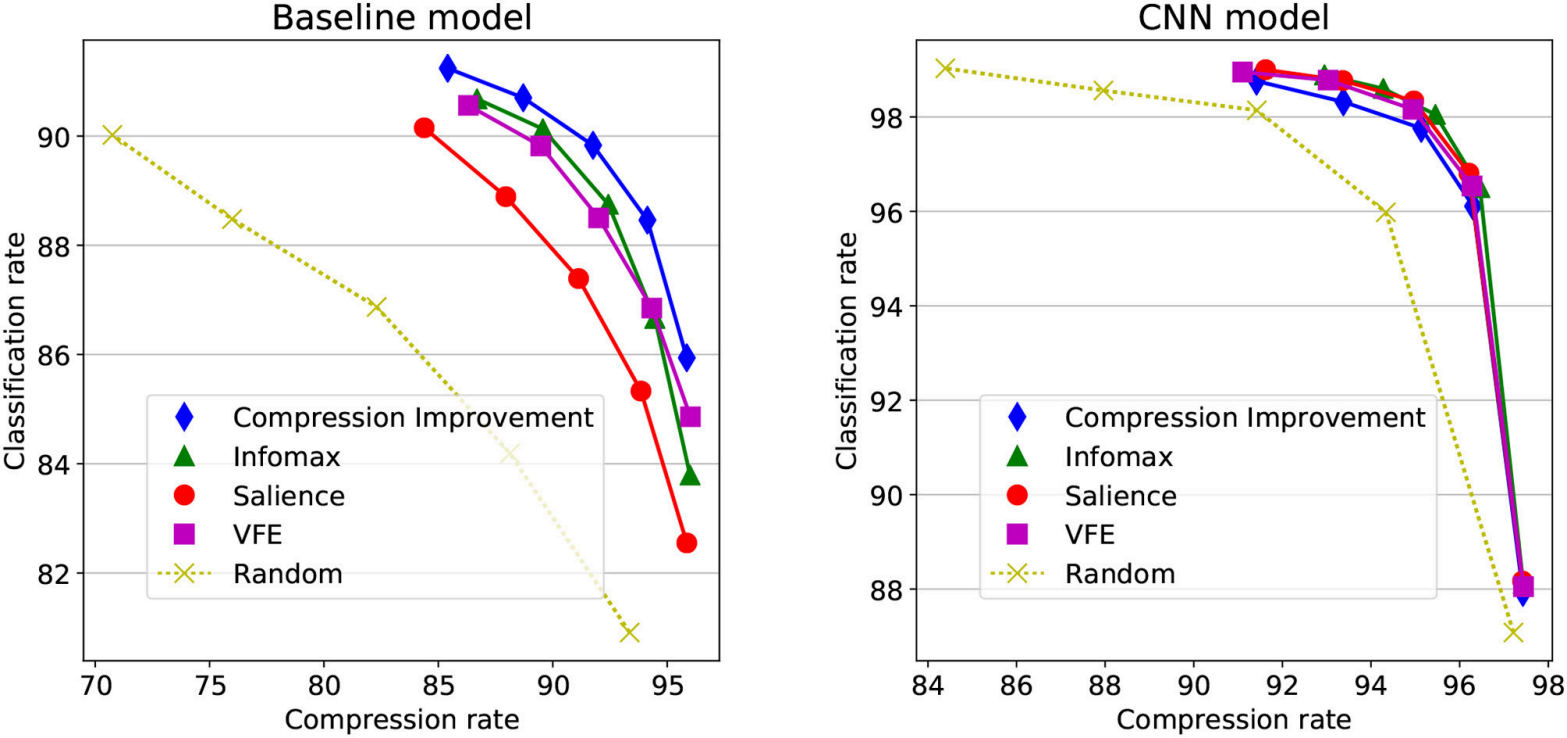
Objective functions comparison. The classification rates and average compression rates are processed after 10,000 sequential scene decoding sessions on the MNIST test set, with different objective (or loss) functions and different values of *H*_ref_. The classification rate is shown in function of the decoding compression rate. **(Left)** Baseline model, with recognition threshold *H*_ref_ varying from 10^−5^ up to $H_{\text{ref}}=10^{-1}$ (from left to right). **(Right)** CNN model, with recognition threshold varying from $H_{\text{ref}}=10^{-2}$ up to *H*_ref_ = 1 (from left to right).

Last, the *decoding compression rate* is defined as the proportion of wavelet coefficients that are bypassed for reaching decision. In Figure 2 first row for instance, a total of 25 coefficient triplets is actually read-out from 7 saccades (not shown), representing about 10% of the total 256 coefficient triplets, issuing a 90% compression rate. The left-hand side of Figure 3 shows how the classification rates vary in function of the average compression rate, for different objective functions and recognition threshold $H_{\text{ref}}\in \left\{10^{-1},10^{-2},10^{-3},10^{-4},10^{-5}\right\}$. The objectives are also compared with a random baseline policy. The classification rates monotonically increase with a *decreasing* recognition threshold. Considering 92% as the upper bound here, a near optimal recognition rate is obtained at $H_{\text{ref}}=10^{-5}$ for the CI objective. Though all objectives functions show a consistent increase of the classification rate with decreasing *H*_ref_, the CI-based policy here overtakes the others policies. The Infomax and the VFE-based policies behave in a close-by fashion, and then the Salience-based policy provides a less effective scene decoding. All scene decoding policies provide elevated compression rates, with a close to optimal classification obtained at around 85% compression of the original data. It must be noticed that still a correct 90% classification rate can be obtained with a random policy at around 70% compression rate, reflecting a strong redundancy in the original images.

##### 3.2.2.2. Convolutional neural network

**Figure 4:**
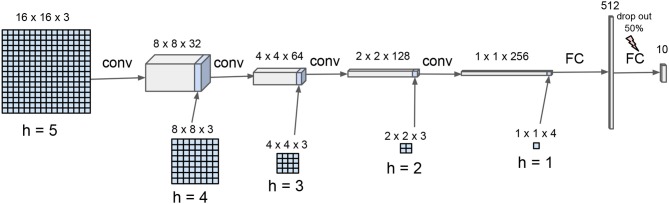
Hierarchical convolutional neural network for scene decoding. The CNN is composed of four convolutional layers and one fully connected layer. The input wavelet hierarchical organization is reflected in scale-dependent input inlay, consistently with stride-2 convolutional spatial integration.

A convolutional neural network (CNN) was designed in order to provide a more effective inference and facilitate comparison with state-of-the-art classifiers (see Figure 4). It is made of five convolution layers having each a distinct input corresponding to the five hierarchical levels of the wavelet decomposition. The CNN is biasless, uses a (2,2) stride for the convolutions *without max-pooling*, promoting *neighbour independence* in the convolutional computation track. Rectified Linear Units are used in all layers, except for the final layer owning linear units.

The network was trained during about 10^6^ epochs with Tensorflow^4^ on a laptop. Sparse foveal-consistent inputs are used for the training. For each training example, many gaze orientation (*i*_1_, *j*_1_), …, (*i*_*n*_, *j*_*n*_) are chosen at random, mimicking a *n*-views foveal visual scan, with *n* randomly set from interval {1, .., 256}. The multi-level input maps are then fed with the corresponding wavelet coefficients triplets, “pyramidally” distilled from *h* = 5 to *h* = 0. The final network is expected to perform recognition on *randomly compressed* images, for which some wavelet coefficients are kept and some wavelet coefficients are discarded. With standard parameter tuning [Adam optimizer – Kingma and Ba (2014) – with a learning rate equal to 10^−4^], the network attains a 99% recognition rate on the test set with non-compressed wavelet transformed inputs (full information case).

The cross-entropy loss used in training allows to interpret the network output as approaching the data log-likelihood (up to a constant), i.e., CNN(***x***_*i, j*_) ≃ log*p*(***x***_*i, j*_|*Z*, (*i, j*)) + *c*. For decoding a scene, the input layers are initialized at zero and progressively filled with new wavelet coefficients obtained during the scene exploration. The output is updated by adding supplementary data at the input only, complementing the data that was previously read, with exp[CNN(***x***^(1:*n*)^|***u***^(1:*n*)^)] ∝ *p*(***x***^(1:*n*)^|*Z*, ***u***^(1:*n*)^) ∝ *p*(*Z*|***x***^(1:*n*)^, ***u***^(1:*n*)^) = *q*^(*n*)^. The posterior update is thus implemented *from the data*. There is no recurrence, sequential accumulation or memory implemented in the network (like in Equation 6).

Following algorithm 2 with the CNN as the approximate cumulated posterior estimator, the decoding efficacy is shown for different objective functions on the right-hand side of Figure 3, with *H*_ref_ ∈ {1, 0.3, 0.1, 0.03, 0.01}. It is to be noted the CNN is only used for estimating the *q*^(*n*)^'s posterior distributions, with the baseline Bernouilli-gated multivariate Gaussian model (Equation 37) used on the predictive/generative side. A clear decoding improvement is obtained when compared with the left-hand side of Figure 3, with higher classification rates with less signal, attaining about 98,8% correct classification with less than 8% of the original image. Still, the general good performances of the decoder blurs the differences between the different policies. All objectives appear here equally good at effectively decoding the scene (except for the random action selection policy).

##### 3.2.2.3. Faulty model and failure robustness

**Figure 5:**
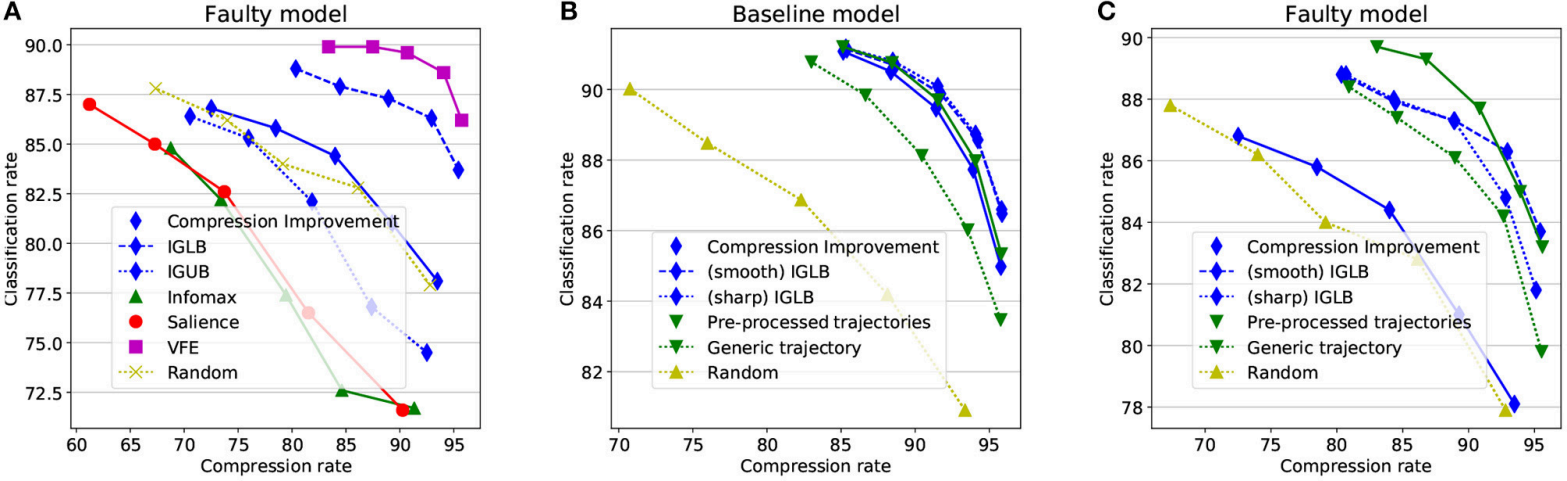
Method comparisons. **(A)** Objective functions comparison in a faulty model (see Figure 3). **(B)** Information Gain-based computational schemes compared on the baseline model. **(C)** Information Gain-based computational schemes compared on the faulty model. The recognition threshold varies from $H_{\text{ref}}=10^{-5}$ up to $H_{\text{ref}}=10^{-1}$ (from left to right).

Predictive policies are known to heavily rest on the generative models, that makes them sensible to model flaws. Resistance to model flaws is thus a property that should be prioritized when acting in unknown or coarsely modeled environments, or in the course of learning. In contrast with CNN-based optimal decoding, a failed probabilistic model was designed by simply setting ρ_*u, z*_ = 1 in Equation (37). This tends to overestimate the signal strength at high frequencies, predicting a dense signal in effectively sparse regions. The classification accuracies are presented on Figure 5A for the different objective functions considered here. In complement to the Compression Improvement (Equation 26), the two variants referred as the IG Lower Bound (Equation 30) and the IG Upper Bound (Equation 35) are also considered.

The faulty model allows here to nicely separate the different metrics with regards to their optimistic vs. conservative flavor. While the CI is here barely better than a random sampling, its conservative and optimistic variants, respectively, do clearly better and clearly worst than random exploration. The VFE loss and the Saliency objectives, as expected, amplify this effect with a strong robustness to model flaws for the VFE loss and, at reverse, a strong sensitivity to model flaws for the Saliency objective. The Infomax also falls here in the optimistic category for its blindness to sequential consistency makes it update the posterior the wrong way.

#### 3.2.3. Scaling Up IG Computation

The scaling of the model needs to be addressed when large control spaces are considered. All predictive policies rely on a mixed encoding setup that implies to consider all ***u***'s and all ***z***'s in the prediction, which scales like $O\left(|U|\times |Z{|}^{2}\right)$ when the predicted posterior is needed in the objective/loss calculation, which is the case for the Infomax, the Saliency, the VFE the CI and the IGUB (algorithm 1), and O(|U|×|Z|) in the IGLB case for it can bypass the post-sample posterior calculation. A quadratic cost may still be considered too heavy in real-case applications, implying to consider cheaper setups.

**Figure 6:**
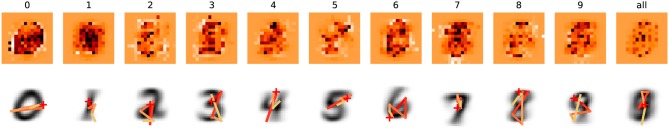
Action maps and pre-processed trajectories. **Upper row** (except rightmost map) color-coded pre-processed guess-consistent action maps for $\hat{\text{z}}\in \left\{0,..,9\right\}$, and ***u***∈{0, .., 15}^2^, using the baseline generative model, from low (whitish) to high (brownish) log posteriors. **Upper row, rightmost map:** Class-independent expected IGLB map. **Lower row:** Corresponding pre-processed visual scan-path (the red “+” provides the initial gaze orientation). Only the 5 first saccades are shown, with average class prototype in the background. The rightmost background image is the average over all classes.

- A first simplification, referred as the “sharp” IGLB in Figures 5B,C, only samples a single ***z*** from *q*^(*n*−1)^, i.e.
  $$\hat{z}=\underset{z}{\text{argmax }}q^{\left(n-1\right)}(z)$$
  with $\hat{z}$ the current *guess*, making the predictive policy scale like O(|U|) for the main loop of algorithm 1 is now over the ***u***'s only.
- An additional simplification can be obtained when considering the IGLB objective alone (Equation 30), for it is, on contrary to all other objectives, independent of the context *q*^(*n*−1)^. For a given model *p*, and for every guess $\hat{z}\in Z$, all the predictive log posteriors $\log p\left(\hat{\text{z}}|{\hat{\text{x}}}_{ẑ,u},\text{u}\right)$ can be pre-processed using the *mode* of the predicted visual field ${\hat{\text{x}}}_{ẑ,u}=\underset{\text{x}}{\text{argmax}}p\left(\text{x}|\hat{\text{z}},\text{u}\right)$ as a sample. This results in a set of class-consistent *action maps* providing, for each u∈U, the expected log posterior value $\log p\left(\hat{\text{z}}|{\hat{\text{x}}}_{ẑ,u},\text{u}\right)$, given $\hat{\text{z}}$ (Figure 6, first row). Then, for each guess $\hat{\text{z}}$, a class-specific visual exploration strategy can be *pre-processed*, following a log-posterior descending order over the ***u***′*s*, from higher IGLB values of toward lower IGLB values (brownish toward whitish in Figure 6). |Z| saccade trajectories of size |U| are then calculated offline and stored in ordered lists, with a O(|U|×|Z|) memory load but only ≃ *O*(1) readout computational cost. In practice, the viewpoint selected at step *n* depends on the current guess $\hat{\text{z}}$, with on-the-fly trajectory switch if the guess is revised during the course of saccades. This strategy is referred a the *pre-processed trajectories* in Figures 2, 5B,C.
- For comparison, a *generic* trajectory was also computed using
  (38) $$\bar{\text{IGLB}}(u)=E_{z\sim p(Z)}\left[\log p(z|{\hat{x}}_{z,u},u)\right]$$
  with a uniform prior over the ***z***′*s*, i.e., $p\left(\text{z}\right)=\frac{1}{\left|Z\right|}$. It is referred a the *generic trajectory* in Figures 2, 5B,C.This strategy is useful in the absence of a definite guess (uniform initial prior for instance).

The action maps allow to analyze in detail the class-consistent orientations (that appear brownish) as opposed to the class-inconsistent orientations (pale orange to white). First to be noticed is the relative scarceness of the class-consistent orientations. A small set of saccades is expected to provide most of the classification information while the rest of the image is putatively uninformative (or even misleading if whitish). A second aspect is that the class-relevant locations are all located in the central part of the images, so there is very few chance for the saccades to explore the periphery of the image where little information is expected to be found. This indicates that the model has captured the essential concentration of class-relevant information in the central part of the images for that particular training set.

The different simplification strategies are compared in Figures 5B,C over the baseline and the faulty models. Both the sharp IGLB and the pre-processed trajectories are shown consistent with the CI objective on Figure 5B, despite their considerably lower computational cost, while the generic trajectory strategy appears less effective. Interestingly, those computational simplifications also remain valid when robustness to model flaws is considered (Figure 5C). Both the sharp IGLB and the pre-processed trajectories allow to reach both robustness and effective classification rates at considerably lower cost than the “smooth” IGLB.

## 4. Conclusion

Stemming from the fovea-based scene decoding problem, a generic *predictive* action selection framework was presented which, accordingly with (Najemnik and Geisler, 2005), rests on a predictive accuracy metric to choose action. An “active” inference approach is also considered, which, accordingly with Friston et al. (2012), optimizes sensory samples selection through action. In our case, the visual field is interpreted under a *mixed emission* model for the visual data is both generated by the viewpoint and the scene constituents. This allows to unify the many objective functions proposed in the literature under a single metric referred as the Compression Improvement (CI) in Schmidhuber (2007), that is shown to provide a consistent interpretation for most of the objective functions used in perception-driven control.

Two variants of the CI objective are then proposed, using either the pre-sample or the post-sample posterior in the approximation. In the pre-sample case, it is shown to be an Information Gain Lower Bound objective that always underestimate the actual Information Gain. The IGLB objective is said conservative for it should prevent from searching for conflicting visual data that may challenge the current interpretation. On the other hand, it is expected to lower the risk of failed interpretation in the case of a (erroneous) conflicting predictions. Conversely, in the post-sample case, the approximate CI is shown to always *overestimate* the actual Information Gain, making it the Information Gain Upper Bound objective (IGUB)—or “optimistic” IG objective. Following the IGUB is expected to perform a more thorough scene exploration for it may preferentially head toward conflicting visual data that may challenge the current interpretation. On the other hand, it is also expected to increase the risk of failed interpretation in the case of (erroneous) conflicting predictions.

Remarkable is the fact that both the Saliency objective, the Infomax objective and the Free Energy epistemic cost, that are classic metrics of the literature, show a consistent inclusion in a more general approximate Information Gain maximization principle. Using for instance the Variational Free Energy (Friston et al., 2015) as a loss (instead of the IGLB) is expected to *bias* the action selection in an even more conservative way. Conversely, using the Saliency objective (Itti and Baldi, 2005) instead of the IGUB is expected to *bias* the action selection in an even more optimistic way, subsequently increasing the risk of a failed scene interpretation.

The presented numerical experiments thus highlight different aspects of the setup. A first and principal result is that state-of-the-art recognition rates are obtained with sequential fovea-based computation using less than 10% of the original signal. This strong input compression is made possible for the visual data owns lot of redundancies that are not used at their best in computer vision, doing useless computations over large parts of the visual scene. The satisfactory results obtained in that case reflect the advantage of mixing a predictive controller with accurate state-of-the-art predictors, here a deep neural network.

A second result is the sub-optimality of many action selection metrics used in literature, like the “Infomax” (Butko and Movellan, 2010) and the “Salience” objectives (Itti and Baldi, 2005), when the scene decoding setup is considered. Their sub-optimality is not manifest with finely-tuned generative models, but becomes patent when a coarse of faulty model is used. This may appear counter-intuitive at first sight for the Infomax objective is vastly dominant in predictive control (Najemnik and Geisler, 2009), while the Salience objective provides among the best predictions for human fixation zones (Itti and Baldi, 2005). The mixed performances of the Salience objective in predictive control may however be attenuated when learning is considered. Heading toward inconsistently modeled places is indeed a sensible behavior when the model is immature. This entails maximizing predictions errors, which is a relevant principle long considered in sparse reinforcement learning (Schmidhuber, 1991; Oudeyer and Kaplan, 2008; Pathak et al., 2017). This trade-off reflects a more general contradiction between exploiting at best the current knowledge from past observations vs. challenging the current interpretation to leverage conflicting facts, a variant of the exploration/exploitation trade-off.

Last, a notorious drawback of the predictive setup is its computational cost scaling with the size of the actions sets, that may grow combinatorially fast with increasing degrees of freedom. Real-world predictive control is thus in need for computationally-effective predictive models, here attainable with the Information Gain Lower Bound (IGLB) objective, that, though maximizing the Information Gain in approximation, allow for low-complexity calculation when replacing the exact posterior with a single guess in the prediction. In discrete latent spaces, it is thus possible to pre-process guess-specific offline trajectories, allowing to bypass computationally-demanding predictions. This strongly simplified setup is shown efficient in our case, showing both competitive decoding compression rates and good robustness to model flaws.

IG-driven fovea-based sequential processing may finally be useful in the case of high dimensional input data (like in e.g., computer vision), and should be tested on more challenging computer vision setups. It is also to be determined how far IG-based action selection may extend to more general partially observed environments, and whether they could challenge more established actions selection strategies in open-ended control setups.

## Author Contributions

The author confirms being the sole contributor of this work and has approved it for publication.

### Conflict of Interest Statement

The author declares that the research was conducted in the absence of any commercial or financial relationships that could be construed as a potential conflict of interest.

## Footnotes

^1^or negative epistemic value.

^2^It is to be noticed that the Kullback-Leibler divergence is here absorbed in the general expectation over the ***x***'s and the ***z***'s.

^3^http://yann.lecun.com/exdb/mnist

^4^https://www.tensorflow.org
