# Supplementary material for: Active Fovea-Based Vision Through Computationally-Effective Model-Based Prediction
Source: Front Neurorobot. 2018 Dec 14;12:76. doi: 10.3389/fnbot.2018.00076 (PMC6302111; doi:10.3389/fnbot.2018.00076)
Supplement: Supplementary file 1 [file Presentation_1.pdf]

Appendix

A Simplified generative model

(i) Emitters independence A first assumption is that two transition processes are independent, i.e. $\text{Pr}(Z, U) = \text{Pr}(Z)\text{Pr}(U)$ so that:

$$(\boldsymbol{z}, \boldsymbol{u}) \sim \text{Pr}(Z, U|\boldsymbol{a}, \boldsymbol{z}_0, \boldsymbol{u}_0) = \text{Pr}(Z|\boldsymbol{a}, \boldsymbol{z}_0, \boldsymbol{u}_0)\text{Pr}(U|\boldsymbol{a}, \boldsymbol{z}_0, \boldsymbol{u}_0)$$

(ii) End-effector control An additional assumption is that the controlled transition process is relatively "fast" in comparison with the uncontrolled one (for, e.g, saccades can be realized in a 100-200 ms interval). In consequence we assimilate the motor command $\boldsymbol{a}$ with a setpoint (or posture) $\boldsymbol{u}$ in the actuator space, that is supposed to be reached at short notice by the motor apparatus once the command is emitted, under classical stability/controllability constraints. This entails that, consistently with the 'end-effector" ballistic control setup (Mussa-Ivaldi and Solla, 2004), $\boldsymbol{u}$ *is independent from* $\boldsymbol{u}_0$, i.e.:

$$\boldsymbol{u} \sim \text{Pr}(U|\boldsymbol{a})$$

The motor command $\boldsymbol{a}$ then corresponds to the desired end-orientation of the sensor, here considered as a setpoint in the actuators space, either expressed in actuators or endpoint coordinates (with hardware-implemented detailed effector response function). Under that perspective, the effector acts on the sensors position and orientation so as to achieve a certain perspective (or view) over the external scene, and the controlled emitter $\boldsymbol{u}$ is now called a *viewpoint*.

(iii) Uncontrolled environment The third important assumption is that the motor command $\boldsymbol{a}$ *is not expected to affect the uncontrolled latent emitter* $\boldsymbol{z}$, i.e.

$$\boldsymbol{z} \sim \text{Pr}(Z|\boldsymbol{z}_0)$$

so that $\boldsymbol{z}$ should depend only on the external dynamics (the external "uncontrolled" process).

(iv) Static assumption Under a scene decoding task, it is rather common to consider the environment as "static" (Butko and Movellan, 2010). This fourth assumption means, in short, that:

$$\text{Pr}(Z|\boldsymbol{z}_0) = \delta(Z, \boldsymbol{z}_0)$$

with δ the Knonecker symbol. The uncontrolled latent emitter $\boldsymbol{z}$ is thus expected to capture all relevant information about the current scene, while remaining invariant throughout the decoding process.

Last, the observation $\boldsymbol{x}$ may rely on both emitters $\boldsymbol{z}$ and $\boldsymbol{u}$, i.e.

$$\boldsymbol{x} \sim \text{Pr}(X|\boldsymbol{z}, \boldsymbol{u}) \tag{39}$$

Each observation $\boldsymbol{x}$ is generated from a mixed emitter $(\boldsymbol{z}, \boldsymbol{u})$, with $\boldsymbol{u}$ the controlled part of the emitter and $\boldsymbol{z}$ the uncontrolled part. Note that $\boldsymbol{z}$ is said the latent state out of habit, though both $\boldsymbol{u}$ and $\boldsymbol{z}$ contribute to the generation of $\boldsymbol{x}$.

For notational simplicity, we absorb here the execution noise (Van Beers et al., 2004) in the measure process, i.e.: $\boldsymbol{x} \sim \text{Pr}(X|\boldsymbol{z}, U)\text{Pr}(U|\boldsymbol{a})$. Then, by notational abuse, we assimilate in the rest

of the paper $\boldsymbol{u}$ (the controlled emitter) with $\boldsymbol{a}$ (the motor command), so that a single variable $\boldsymbol{u} \equiv \boldsymbol{a}$ should be used for both. Each different $\boldsymbol{u}$ is thus both interpreted as a motor command and as an emitter. As a motor command, it is controllable, i.e. determined by a controller. As an emitter, it monitors the generation of the sensory field, in combination with the latent state $\boldsymbol{z}$.

B Viewpoint-dependent variational encoding setup

The *variational encoding* perspective (Hinton and Zemel, 1994) was originally developed to train unsupervised autoencoder neural networks. If $\boldsymbol{x}$ is the original data, the corresponding code $\boldsymbol{z}$ is generated by a distribution q, i.e. $\boldsymbol{z} \sim q(Z)$. This distribution is called the *encoder*. Then, the reconstruction is made possible with a second conditional probability over the codes, i.e. $p(X|\boldsymbol{z})$, that is called the *decoder*. If $\boldsymbol{z}$ is the current code, the reconstructed data is $\tilde{\boldsymbol{x}} \sim p(X|\boldsymbol{z})$.

In short, the efficacy of a code is estimated by an information-theoretic quantity, the "reconstruction cost" that is defined for every $\boldsymbol{x}$ knowing p and q:

$$F(\boldsymbol{x}) = \mathbb{E}_{z\sim q}\left[-\log(p(\boldsymbol{x}|\boldsymbol{z}))\right] + \mathrm{KL}(q(Z)||p(Z)) \tag{40}$$

$$= -\log p(\boldsymbol{x}) + \mathrm{KL}(q(Z)||p(Z|\boldsymbol{x})) \tag{41}$$

with $p(Z)$ the prior over the latent state. F is also said the Variational Free Energy (VFE), for it shares shares a mathematic analogy with the Helmhotz Free Energy (Friston, 2010). Minimizing the cost F according to p and q thus means minimizing the "surprise" caused by observing the data $\boldsymbol{x}$ (Friston, 2010).

Viewpoint-dependent VFE If we now turn back to the viewpoint selection setup, an additional factor $\boldsymbol{u}$ (the viewpoint) comes into the play. The data $\boldsymbol{x}$ that is actually read is now conditioned on $\boldsymbol{u}$, so that:

$$F(\boldsymbol{x}|\boldsymbol{u}) = \mathbb{E}_{z\sim q}\left[-\log(p(\boldsymbol{x}|\boldsymbol{z},\boldsymbol{u}))\right] + \mathrm{KL}(q(Z)||p(Z)) \tag{42}$$

$$= -\log p(\boldsymbol{x}|\boldsymbol{u}) + \mathrm{KL}(q(Z)||p(Z|\boldsymbol{x},\boldsymbol{u})) \tag{43}$$

When only the variations of p and q are considered in the optimization, each viewpoint $\boldsymbol{u}$ provides a distinct optimization problem that is resolved by finding $q(Z) \simeq p(Z|\boldsymbol{x},\boldsymbol{u})$. Each $\boldsymbol{u}$ may thus drive a different posterior and thus a different reconstruction cost. It is thus feasible to change (and optimize) the reconstruction cost through changing $\boldsymbol{u}$.

Sequential viewpoint-dependent VFE When generalized to many observations: $(\boldsymbol{x},\boldsymbol{u})$, $(\boldsymbol{x}',\boldsymbol{u}')$, ..., $(\boldsymbol{x}^{(n)},\boldsymbol{u}^{(n)})$, the nth reconstruction cost $F^{(n)}(\boldsymbol{x}^{(n)}|\boldsymbol{u}^{(n)},...,\boldsymbol{x},\boldsymbol{u})$ also obeys to the chain rule (see eq. 6), i.e. is estimated from $q^{(n-1)}$, $\boldsymbol{u}^{(n)}$ and $\boldsymbol{x}^{(n)}$ only:

$$F(\boldsymbol{x}^{(n)}|\boldsymbol{u}^{(n)};q^{(n-1)}) = \mathbb{E}_{\boldsymbol{z}\sim q}\left[-\log p(\boldsymbol{x}^{(n)}|\boldsymbol{z},\boldsymbol{u}^{(n)})\right] + \mathrm{KL}(q(Z)||q^{(n-1)}(Z)) \tag{44}$$

$$= -\log p(\boldsymbol{x}^{(n)}|\boldsymbol{u}^{(n)}) + \mathrm{KL}(q(Z)||p(Z|\boldsymbol{x}^{(n)},\boldsymbol{u}^{(n)};q^{(n-1)})) \tag{45}$$

with $q^{(n-1)}$ having the role of the prior, providing a *forward* variational encoding scheme (see also (Chung et al., 2015; Fraccaro et al., 2016).
